# Supplementary material for: Modeling glioblastoma heterogeneity as a dynamic network of cell states
Source: Mol Syst Biol. 2021 Sep 16;17(9):e10105. doi: 10.15252/msb.202010105 (PMC8444284; doi:10.15252/msb.202010105)
Supplement: Supplementary file 6 — Source Data for Figure 5 [file MSB-17-e10105-s004.zip › Figure5A_sourcedata/GSEA_3017/nosigdb_stateF.GseaPreranked.1622795015644/OLSEN_SCPLIKE.html]

Details for gene set OLSEN\_SCPLIKE[GSEA]

|  || Dataset | state33017 |
| Phenotype | NoPhenotypeAvailable |
| Upregulated in class | na\_pos |
| GeneSet | OLSEN\_SCPLIKE |
| Enrichment Score (ES) | 0.5502155 |
| Normalized Enrichment Score (NES) | 2.8584788 |
| Nominal p-value | 0.0 |
| FDR q-value | 0.0 |
| FWER p-Value | 0.0 |
Table: GSEA Results Summary

  

Fig 1: Enrichment plot: OLSEN\_SCPLIKE      
 Profile of the Running ES Score & Positions of GeneSet Members on the Rank Ordered List

  

| PROBE | GENE SYMBOL | GENE\_TITLE | RANK IN GENE LIST | RANK METRIC SCORE | RUNNING ES | CORE ENRICHMENT || 1 | NEAT1 |  |  | 0 | 2.864 | 0.0981 | Yes |
| 2 | CST3 |  |  | 15 | 1.273 | 0.1353 | Yes |
| 3 | LGALS3 |  |  | 16 | 1.261 | 0.1785 | Yes |
| 4 | APOE |  |  | 17 | 1.244 | 0.2211 | Yes |
| 5 | S100B |  |  | 20 | 1.167 | 0.2601 | Yes |
| 6 | PNRC1 |  |  | 23 | 1.070 | 0.2959 | Yes |
| 7 | IFITM3 |  |  | 38 | 0.987 | 0.3233 | Yes |
| 8 | EDNRB |  |  | 44 | 0.957 | 0.3538 | Yes |
| 9 | SPARC |  |  | 52 | 0.929 | 0.3825 | Yes |
| 10 | CRYAB |  |  | 85 | 0.783 | 0.3948 | Yes |
| 11 | MALAT1 |  |  | 90 | 0.773 | 0.4194 | Yes |
| 12 | APP |  |  | 95 | 0.759 | 0.4436 | Yes |
| 13 | IFITM2 |  |  | 103 | 0.749 | 0.4661 | Yes |
| 14 | B2M |  |  | 110 | 0.736 | 0.4885 | Yes |
| 15 | IGFBP7 |  |  | 164 | 0.643 | 0.4865 | Yes |
| 16 | ITM2B |  |  | 181 | 0.610 | 0.5002 | Yes |
| 17 | BTG1 |  |  | 222 | 0.574 | 0.5017 | Yes |
| 18 | PRNP |  |  | 269 | 0.538 | 0.4992 | Yes |
| 19 | GAS7 |  |  | 299 | 0.511 | 0.5036 | Yes |
| 20 | PTPRZ1 |  |  | 310 | 0.505 | 0.5163 | Yes |
| 21 | VAMP5 |  |  | 341 | 0.491 | 0.5195 | Yes |
| 22 | COL4A1 |  |  | 347 | 0.489 | 0.5340 | Yes |
| 23 | SPTBN1 |  |  | 349 | 0.487 | 0.5502 | Yes |
| 24 | PSAP |  |  | 418 | 0.463 | 0.5352 | No |
| 25 | TIMP3 |  |  | 516 | 0.428 | 0.5059 | No |
| 26 | GPM6B |  |  | 553 | 0.413 | 0.5038 | No |
| 27 | PMP2 |  |  | 562 | 0.411 | 0.5142 | No |
| 28 | IGFBP5 |  |  | 564 | 0.410 | 0.5278 | No |
| 29 | CNN3 |  |  | 576 | 0.407 | 0.5367 | No |
| 30 | S100A11 |  |  | 692 | 0.373 | 0.4973 | No |
| 31 | MEF2C |  |  | 696 | 0.372 | 0.5087 | No |
| 32 | KCTD12 |  |  | 808 | 0.345 | 0.4702 | No |
| 33 | AP1S2 |  |  | 884 | 0.329 | 0.4475 | No |
| 34 | DST |  |  | 896 | 0.327 | 0.4537 | No |
| 35 | CD59 |  |  | 897 | 0.326 | 0.4648 | No |
| 36 | IFI6 |  |  | 922 | 0.323 | 0.4650 | No |
| 37 | AHR |  |  | 942 | 0.319 | 0.4673 | No |
| 38 | ITGB8 |  |  | 970 | 0.314 | 0.4658 | No |
| 39 | CD9 |  |  | 1070 | 0.297 | 0.4311 | No |
| 40 | FN1 |  |  | 1238 | 0.275 | 0.3648 | No |
| 41 | CTSD |  |  | 1245 | 0.275 | 0.3715 | No |
| 42 | NRXN1 |  |  | 1374 | 0.259 | 0.3224 | No |
| 43 | ANXA2 |  |  | 1511 | -0.263 | 0.2697 | No |
| 44 | ITGB1 |  |  | 1521 | -0.265 | 0.2747 | No |
| 45 | ZFP36L1 |  |  | 1590 | -0.284 | 0.2536 | No |
| 46 | LGALS1 |  |  | 1799 | -0.362 | 0.1717 | No |
| 47 | LIMA1 |  |  | 1899 | -0.421 | 0.1413 | No |
| 48 | CTNNAL1 |  |  | 2059 | -0.548 | 0.0879 | No |
Table: GSEA details [plain text format]

  

Fig 2: OLSEN\_SCPLIKE: Random ES distribution      
 Gene set null distribution of ES for **OLSEN\_SCPLIKE**
